# Estimated health benefits, costs and cost-effectiveness of eliminating industrial trans-fatty acids in Nigeria: cost-effectiveness analysis

**DOI:** 10.1136/bmjgh-2023-014294

**Published:** 2024-04-17

**Authors:** Matti Marklund, Leopold N Aminde, Mary Njeri Wanjau, Boni M Ale, Adedayo E Ojo, Clementina E Okoro, Abimbola Adegboye, Liping Huang, J Lennert Veerman, Jason HY Wu, Mark D Huffman, Dike B Ojji

**Affiliations:** 1 Department of Public Health and Caring Sciences, Uppsala University, Uppsala, Sweden; 2 Food Policy, The George Institute for Global Health, Newtown, New South Wales, Australia; 3 Department of Epidemiology, Johns Hopkins Bloomberg School of Public Health, Baltimore, Maryland, USA; 4 Welch Center for Prevention, Epidemiology, and Clinical Research, Johns Hopkins University, Baltimore, Maryland, USA; 5 School of Medicine and Dentistry, Griffith University, Gold Coast, Queensland, Australia; 6 Cardiovascular Research Unit, University of Abuja Teaching Hospital, Gwagwalada, Abuja, Nigeria; 7 Holo Healthcare, Nairobi, Kenya; 8 Department of Epidemiology and Global Health, University Medical Centre Utrecht, Utrecht, Netherlands; 9 Federal Capital Territory Primary Health Care Board, Abuja, Nigeria; 10 National Agency for Food and Drug Administration and Control, Abuja, Federal Capital Territory, Nigeria; 11 School of Public Health and Community Medicine, University of New South Wales, Sydney, New South Wales, Australia; 12 Washington University in St Louis, St Louis, St Louis, USA; 13 Department of Internal Medicine, Faculty of Clinical Sciences, University of Abuja, Abuja, Federal Capital Territory, Nigeria

**Keywords:** Health economics, Nutrition, Public Health, Cardiovascular disease

## Abstract

**Introduction:**

Nigeria is committed to reducing industrial *trans-*fatty acids (iTFA) from the food supply, but the potential health gains, costs and cost-effectiveness are unknown.

**Methods:**

The effect on ischaemic heart disease (IHD) burden, costs and cost-effectiveness of a mandatory iTFA limit (≤2% of all fats) for foods in Nigeria were estimated using Markov cohort models. Data on demographics, IHD epidemiology and *trans-*fatty acid intake were derived from the 2019 Global Burden of Disease Study. Avoided IHD events and deaths; health-adjusted life years (HALYs) gained; and healthcare, policy implementation and net costs were estimated over 10 years and the population’s lifetime. Incremental cost-effectiveness ratios using net costs and HALYs gained (both discounted at 3%) were used to assess cost-effectiveness.

**Results:**

Over the first 10 years, a mandatory iTFA limit (assumed to eliminate iTFA intake) was estimated to prevent 9996 (95% uncertainty interval: 8870 to 11 118) IHD deaths and 66 569 (58 862 to 74 083) IHD events, and to save US$90 million (78 to 102) in healthcare costs. The corresponding lifetime estimates were 259 934 (228 736 to 290 191), 479 308 (95% UI 420 472 to 538 177) and 518 (450 to 587). Policy implementation costs were estimated at US$17 million (11 to 23) over the first 10 years, and US$26 million USD (19 to 33) over the population’s lifetime. The intervention was estimated to be cost-saving, and findings were robust across several deterministic sensitivity analyses.

**Conclusion:**

Our findings support mandating a limit of iTFAs as a cost-saving strategy to reduce the IHD burden in Nigeria.

**What is already known on this topic**

- High intake of trans-fatty acids is a recognised risk factor for ischaemic heart disease, leading to the implementation of policies worldwide to eliminate industrial trans-fats.
- In 2023, Nigeria, Africa’s most populous nation, regulated to restrict industrial trans-fatty acids in food, but so far, the regulation has not been implemented.

**What this study adds**

- This study provides quantitative estimates of the cost-effectiveness and potential impact of this policy in Nigeria, indicating it could prevent about 10 000 ischaemic heart disease deaths after a decade, with healthcare cost-savings were estimated to outweigh implementation costs.
- These findings, alongside previous analyses in high-income nations, suggest that eliminating industrial trans-fats can be a cost-effective or even cost-saving strategy for reducing ischaemic heart disease globally.

**How this study might affect research, practice or policy**

- The study provides crucial data for Nigerian policy-makers and those in other low-income and middle-income countries considering trans-fatty acid regulations.
- It underscores the potential benefits of such policies in terms of public health and healthcare cost-savings, supporting their implementation and the fight against ischaemic heart disease across various economic development stages and continents.

## Introduction

In Nigeria and other African countries, the burden of ischaemic heart disease (IHD) is increasing rapidly and in 2019, around 70 000 deaths in Nigeria were attributed to IHD.^1^ Diet plays a key role in the development of IHD, and a high intake of *trans-*fatty acids is a well-established dietary risk factor for IHD. *Trans*-fatty acids are a group of unsaturated fatty acids with one or more double bonds in the *trans* configuration, making the carbon chain straighter compared with *cis-*fatty acids. This structure allows the *trans-*fatty acid molecules to pack more densely and thereby increases their melting point, making them solid at room temperature, in contrast to their cis-counterparts. High intake of *trans-*fatty acids is known to cause atherosclerosis,^2^ and every 2% increase in total energy intake from *trans*-fatty acids is associated with a 23% higher risk of incident IHD.^3^ Although *trans*-fatty acids occur naturally at low levels in meat and milk from ruminants, the predominant source in many countries is industrially made partially hydrogenated vegetable oils (PHVO) in processed foods.^4^

The WHO recommends limiting *trans*-fatty acid intake to <1% energy (%E).^5^ In order to help countries to implement successful strategies to eliminate industrially derived *trans*-fatty acids (iTFA) from the food supply, WHO has developed the REPLACE package which includes six strategic action areas: (1) review dietary sources of iTFA and the landscape for required policy change; (2) promote the replacement of iTFA with healthier oils and fats; (3) legislate or enact regulatory actions to eliminate iTFA; (4) assess and monitor *trans*-fatty acid content in the food supply and changes in trans-fatty acid consumption in the population; (5) create awareness of the negative health impact of *trans*-fatty acids among policy-makers, producers, suppliers and the public; and (6) enforce compliance with policies and regulations.^6^ An increasing number of countries have implemented strategies to reduce iTFA in the food supply.^7 8^ Such policies can range from voluntary reformulation, mandatory labelling, to banning iTFA or PHVO entirely.^9^ The global progress in limiting iTFA through policy is monitored by the WHO.^10^ Although several African countries (including Nigeria) have expressed commitment to reduce iTFA from the food supply, before 2023, only South Africa had implemented a best-practice policy (ie, mandatory limit of iTFA ≤2% of all fats) as defined by the WHO in the African continent.^10^

In April 2023, Nigeria passed regulations stating that ‘Fats, oils and foods containing fats and oils intended for human consumption of which the content of trans-fat exceeds 2 g per 100 g of fat or oil are prohibited in Nigeria’. Quantitative estimates of the potential health and economic impact of such regulation could aid its implementation in Nigeria and may also guide policy-makers in other low-income and middle-income countries, in Africa and elsewhere. Thus, we conducted a modelling study to estimate the cost-effectiveness of a mandatory limit on iTFA content (≤2% of all fats) in the Nigerian food supply, considering policy costs, IHD burden and healthcare expenditures. We hypothesised that a limit of iTFA in the food supply would be a cost-effective strategy to reduce the IHD burden in Nigeria.

## Methods

### Study design

Using a multiple cohort proportional multistate life table (Markov) model, we estimated the impact on health outcomes and related costs of an iTFA limit (≤2% of all fats) for the Nigerian food supply. This limit was expected to cover all foods and ingredients containing iTFA. The modelling framework, which has been employed to evaluate similar policies for Australia and Kenya,^11 12^ estimated IHD-related outcomes and total healthcare costs resulting from the intervention, where the life table method transmits changes in iTFA intake to IHD-related morbidity and mortality in the modelled population. In this study, we modelled the adult Nigerian population (≥20 years) in 5-year female and male cohorts, where each cohort was simulated until all individuals died or reached 100 years of age. We compared all outcomes estimated for a reference population with a stable *trans*-fatty acid intake comparable to the intake of the Nigerian population before the intervention, and an intervention population with identical characteristics but lower *trans*-fatty acid intake due to the elimination of iTFA from the food supply. The difference between the reference and intervention populations was calculated for IHD incidence and deaths, life years and health-adjusted life years (HALYs). Results were reported for time horizons of 5 years, 10 years and the population lifespan (ie, the time from policy implementation until all individuals died or reached 100 years of age). In this study, we used an extended healthcare perspective to include implementation costs for government and industry, which are directly related to the intervention. We inflated costs and HALYs to 2019 values and used a 3% discount rate in the main analysis, as recommended by the first and second Panels on Cost-Effectiveness in Health and Medicine.^13 14^ Key inputs and assumptions are presented in table 1, whereas the Consolidated Health Economic Evaluation Reporting Standards 2022 (CHEERS 2022) checklist and the original study proposal and analytical plan are available in online supplemental appendices 1-2. While the present study shares the same framework and methodology as our recent cost-effectiveness analysis of an iTFA policy in Kenya,^12^ there are important differences in how key inputs were derived, particularly in terms of costs, as described below.

**Table 1:** Key input data and assumptions

Input	Stratification	Values	Source	Note
Preintervention TFA intake, E%	Age, sex	Online supplemental table S1	2019 Global Burden of Disease (GBD) study	For each model iteration, random draws from age-sex-specific lognormal TFA distributions were made.
Postintervention TFA intake, E%	n/a	0 (primary analysis) Mean ± SD: 0.1±0.01 (sensitivity analysis)	2018 Global Dietary Database (estimates of dairy intake)	Given the minimal intake of naturally occurring trans-fatty acids (eg, from dairy) compared to countries like Australia, UK and Denmark, the intervention was assumed to virtually eliminate trans-fatty intake in Nigeria. However, we explored the impact of a low but existing TFA intake post intervention in a sensitivity analysis.
Theoretical minimum risk distribution of TFA intake, E%	n/a	0	Marklund *et al,* Plos Med 2020	The theoretical minimum risk distribution of TFA intake was assumed to equal the intake of natural occurring TFA.
Population size	Age, sex	Online supplemental table S2	2019 Global Burden of Disease (GBD) study	
Mortality rate	Age, sex	Online supplemental table S2	2019 Global Burden of Disease (GBD) study	
IHD incidence, prevalence and case fatality rates	Age, sex	Online supplemental table S3	2019 Global Burden of Disease (GBD) study	
Disability weights	Age, sex	Online supplemental tables S4 and S5	2019 Global Burden of Disease (GBD) study	
Healthcare costs	Age, sex	Online supplemental table S6	Rosendaal *et al*, PLOS ONE 2016.	We used cost estimates of acute care after an IHD event per patient (ie, incident IHD costs) and costs of follow-up care after an IHD event per patient per year (ie, prevalent IHD costs) from Rosendaal *et al*^20^ and estimates of total health expenditure from the Nigerian National Health Accounts for 2017 report.^22^ In the absence of a Nigerian source, we derived age and sex distributions of total health expenditure in Nigeria by assuming the relative expenditure by age and sex from the Kenya Household Health Expenditure and Utilisation Survey, 2013. The total health expenditure was then divided by population numbers in each sex and age-group to derive the per capita expenditure. Non-IHD costs were calculated for each age-sex group, subtracting IHD costs from total healthcare expenditure for that group, and was then divided by the group’s population size to estimate per capita non-IHD costs. All healthcare costs were inflated to 2019. For each model iteration, random draws from triangular distributions of IHD costs and normal distributions of non-IHD costs (assuming SD equal to 20% of central estimates) were made.
RR for IHD per 2%E from TFA	Age	Online supplemental table S7	Afshin *et al*, Lancet 2019	For each model iteration, random draws from age-specific lognormal RR distributions were made.
Government policy implementation costs	n/a	Online supplemental table S8	NAFDAC estimates	The following costs were considered: national legislation costs; costs for NAFDAC (ie, work force expenses, enlightenment activities, enforcement activities, workshops activities, monitoring, and other activities including research); FMoH costs for legislation; SON costs for revising food standard regulations; and CPC costs for enlightenment activities. For each model iteration, random draws from normal distributions of implementation costs were made, assuming SD equal to 20% of central estimates.
Industry reformulation	n/a	Online supplemental table S8	Marklund *et al*, Plos Med, 2020	Reformulation costs were calculated using equivalent USD costs from UK estimates (25 000 GBP per product)^5^ multiplied by the number of products in the Nigerian food supply potentially containing industrial TFA (primary model: n=331; sensitivity analysis: n=662). Annual cost to industry equaling 1% of the initial reformulation cost was assumed. For each model iteration, random draws from normal distributions of initial and annual reformulation costs were made, assuming SD equal to 20% of central estimates.

Table created by the authors.

CPC, Consumer Protection Commission; E%, energy percentage; FMoH, Federal Ministry of Health; GBP, British pound; IHD, ischaemic heart disease; n/a, not available/applicable; NAFDAC, National Agency for Food and Drug Administration and Control; RR, relative risk; SON, Standard Organisation of Nigeria; TFA, trans-fatty acid; USD, United States dollar.

### Data sources

From the 2019 Global Burden of Disease (GBD) study, we derived the mean and SD of baseline *trans*-fatty acid intake, expressed as energy percentage (%E), separately for n=30 specific age-sex groups (online supplemental table S1). We modelled the effect of a mandatory limit of iTFA to ≤2% in all fats, oils and foods. Similar to our previous cost-effectiveness analysis of a comparable policy in Kenya,^12^ we assumed that the policy would virtually eliminate intake of total *trans*-fatty acids in all age-sex groups (ie, postintervention mean intake=0%E) in the primary analysis, as the mandatory limit has been effective in eliminating iTFA from the food supply in other countries (eg, Denmark^15^), and the intake of naturally occurring *trans-*fatty acids (ie, from meat and dairy from ruminant animals) is likely negligible in Nigeria (table 1).^16 17^

Estimates of population size and overall mortality rate, as well as IHD-specific estimates of incidence, prevalence, mortality and years lived with disability were obtained from the GBD 2019 study (online supplemental appendix and online supplemental appendix and tables S2-S5).^18^ We used the DisMod II software^19^ to obtain estimates of case fatality rates for IHD, and we calculated IHD disability weights to be used for the estimation of HALYs (online supplemental appendix and tables S3-S5).^12^

We derived estimates of the cost of incident IHD and prevalent IHD from a study by Rosendaal *et al*,^20^ and as previously described,^12^ estimated total, IHD-related and non-IHD-related health expenditure stratified by age and sex (online supplemental appendix and table S6).^12^ To estimate government policy implementation costs, local experts including clinicians, academics and Ministry of Health and National Agency for Food and Drug Administration and Control (NAFDAC) staff were contacted to determine the cost components and estimates that would be required to implement a mandatory limit of iTFA among foods in Nigeria (online supplemental appendix). The government costs included: legislation costs (ie, preparing the law at the Federal Ministry of Health and passing the bill at the National Assembly); NAFDAC costs which included human resources (workforce per geopolitical region, media and enlightenment activities, enforcement and monitoring, other activities, eg, research); Standard Organization of Nigeria costs for revising food standard regulations; and Consumer Protection Commission costs for consumer information campaigns. These cost estimates were derived from the cost of similar activities carried out in previous years, with information sourced from the Finance and Accounts departments of the various relevant ministries, departments, and agencies (MDAs). We used an activity-based budgeting system as our costing framework. This costing framework is normally adopted by all MDAs in developing their annual work plan. It highlights the activities to be carried out and costs them using prevailing market prices. The legislative cost was equivalent to the cost for the various agencies.

We estimated industry costs for reformulation as previously described,^11 12^ based on our recent food supply analysis, where we identified 310 packaged food products potentially containing iTFA in the Nigerian supermarkets (online supplemental appendix).^21^ All costs were inflated to 2019 values using World Bank consumer price indices for Nigeria.^22^

### Statistical analysis

#### Estimation of health benefits and cost-effectiveness

To estimate the proportional change in IHD incidence due to the elimination of iTFAs, we used the reference and intervention *trans*-fatty acid intakes and the relative risk of IHD per %E of *trans*-fatty acid intake to calculate the potential impact fraction (PIF),^11 12^ according to Barendregt’s continuous ‘distribution shift’ PIF method (online supplemental appendix and table S7).^23^ The PIF was used to calculate the effect on IHD incidence due to the reduction in trans-fatty acid intake (online supplemental appendix), and the estimated incidence rates were used in the disease Markov model to calculate reference and intervention IHD prevalence and mortality. The changes in IHD mortality rate are then entered into the lifetable to alter the overall mortality rates and recalculate the life years. To account for time spent in suboptimal health due to IHD and any other conditions present, we calculated HALYs using estimates derived from prevalence and years lived with disability from IHD and other causes. One HALY thus represents the equivalent of a year in perfect health. HALYs gained were calculated as the difference in HALYs between the reference and intervention populations. Changes in healthcare expenditures were estimated both for IHD-related healthcare and total healthcare. The change in IHD-related healthcare expenditure was based on the predicted reduction in IHD mortality and morbidity. Overall healthcare costs in added years of life were also included.^24^ Impact on health outcomes (ie, HALYs gained and averted or postponed IHD events and deaths) and healthcare cost-savings were estimated over the total population and separately for women and men.

Net costs included policy costs and healthcare costs (including costs unrelated to IHD) and were used to calculate the incremental cost-effectiveness ratios (ICERs), defined as the difference in net costs of the intervention compared with current practice, divided by the difference in HALYs. We used the Nigeria-specific cost-effectiveness threshold described by Pichon-Riviere *et al*,^25^ which defines a cost-effective intervention as ICER <US$374 per HALY gained. A negative net cost indicated a cost-saving intervention. The costs were estimated in US dollars and converted to local currency (Naira, ₦) using the average exchange rate of 1 July 2019 (US$1= ₦358).

#### Uncertainty and sensitivity analysis

As previously described,^11 12^ we quantified the parameter uncertainty around the modelled estimates in Monte Carlo simulations (n=2000). For each iteration, a draw was made from the distributions of *trans-*fatty acid intake, relative risks, healthcare costs and policy implementation costs. The point estimate and 95% uncertainty intervals (UI) were defined as the mean and 2.5th–97.5th percentiles, respectively, of the distribution of the intervention effects (eg, HALYs gained) estimated across all 2000 iterations using the Ersatz V.1.35 software. Similarly, Monte Carlo simulations (n=2000) of policy implementation costs were conducted in RStudio V.1.1.423. For each simulation (n=2000), we calculated ICER and return on government investment (defined as healthcare cost-savings divided by government implementation costs). The net costs and ICERs of all simulations were used to estimate the probability of the policy being cost-saving and cost-effective.

Similar to our previous cost-effectiveness analysis of an iTFA policy in Kenya, we used univariate sensitivity analyses to explore the impact of variation in discount rates (0% and 6%), *trans-*fatty acid intake and policy implementation costs (table 1). We also evaluated the impact of 50% lower or higher mean and SD of preintervention intakes. To test the effect of a potentially greater prevalence of iTFA among Nigerian foods (eg, due to iTFA-containing foods not being included in the large nutrition composition database from which the estimate of iTFA-containing foods was based on), the number of products potentially containing iTFA was assumed to be twice as many as identified in the database.^21^ Experience of iTFA regulations in Denmark has suggested negligible reformulation costs^26^ and reformulation can be considered to be a part of the natural life cycle of a product.^27^ Thus, we conducted a sensitivity analysis assuming no industry costs. We also evaluated the impact of 50% greater monitoring costs compared with our primary model.

To evaluate the potential impact of partial elimination of iTFA in the Nigerian food supply (eg, due to suboptimal compliance to the mandatory iTFA limits), we conducted a threshold analysis to estimate the HALYs gained, the net cost, and the ICER in scenarios with gradually greater reductions in iTFA intake (from 0.05%E to 0.20%E lower intake compared with the base population).

#### Patient and public involvement

No patients were involved in setting the research question or the outcome measures, nor were they involved in developing plans for design or implementation of the study. No patients were asked to advise on interpretation or writing up of results. An author reflexivity statement is available as an online online supplemental appendix.

## Results

### Health impact

**Figure 1:**
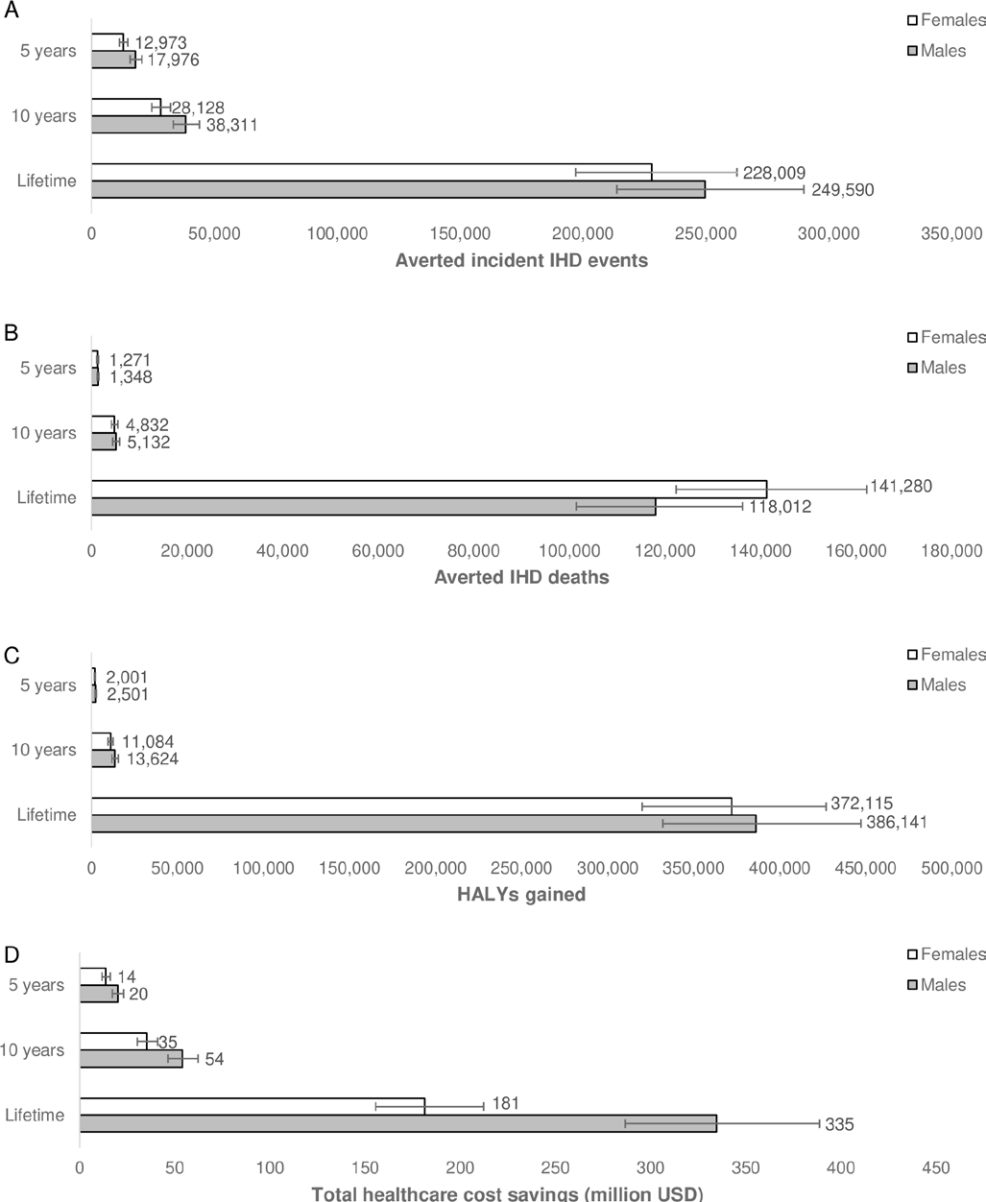
Sex-specific estimates of averted ischaemic heart disease (IHD) events (A), averted IHD deaths (B), health-adjusted life years (HALYs) gained (C) and total healthcare cost-savings (D) after 5 years, 10 years and over the population lifetime. Data labels indicate mean of n=2000 simulations and error bars represent 95% uncertainty intervals. Figure created by the authors.

In the first 10 years, a mandatory limit of iTFA content in the Nigerian food supply was estimated to avert or postpone ~67 000 incident IHD events and ~10 000 IHD deaths, compared with a base case scenario maintaining current intake of *trans*-fatty acids (table 2). Around 480 000 IHD events and ~260 000 IHD deaths could be averted over the population’s lifetime. The intervention could generate some 25 000 HALYs over the first 10 years, and ~760 000 HALYs over the population’s lifetime. The estimated deaths and incidences averted and HALYs gained were evenly distributed among men and women (figure 1), especially for the lifetime horizon.

**Table 2:** Estimated health and health economic effects of a mandatory limit of iTFA content (≤2% of all fats) in the Nigerian food supply, assumed to eliminate TFA intake (ie, postintervention TFA intake = 0%E)

	5 years	10 years	Population lifetime
IHD incidence			
n	−31,018 (−34 571; −27 488)	−66 569 (−74 083; −58 862)	−479 308 (−538 177; −420 472)
%^‡^	−3.51 (−3.91; −3.14)	−3.42 (−3.81; −3.05)	−2.21 (−2.49; −1.94)
IHD deaths			
n	−2625 (−2914; −2334)	−9996 (−11 118; −8870)	−259 934 (−290 191; −228 736)
%^‡^	−0.76 (−0.85; −0.68)	−1.32 (−1.47; −1.18)	−2.20 (−2.47; −1.94)
Health-adjusted life years, n^§^	4515 (3995; 5021)	24 774 (21 888; 27 643)	759 831 (668 211; 851 217)
IHD-related healthcare costs, million USD^§^	−35.9 (−40.8; −31.3)	−100.6 (−113.3; −87.9)	−749.0 (−843.0; −657.3)
Total healthcare costs, million USD^§^	−34.0 (−38.8; −29.6)	−89.6 (−101.6; −77.9)	−518.0 (−587.2; −450.4)
Total implementation costs, million USD^§^	15.6 (9.8; 21.1)	17.2 (11.2; 23.0)	26.2 (19.0; 33.2)
Government implementation costs, million USD^§^	0.89 (0.80; 0.97)	1.96 (1.81; 2.12)	7.81 (7.54; 8.09)
Industry reformulation costs, million USD^§^	14.7 (9.0; 20.3)	15.3 (9.3; 21.1)	18.4 (11.2; 25.4)
Net costs, million USD^§^	−18.5 (−25.7; −11.1)	−72.4 (−85.4; −59.5)	−491.8 (−561.0; −427.0)

Vaues are mean (95% uncertainty interval) derived from n=2,000 Monte Carlo simulations.

† Table created by the authors.

‡ Expressed as a percentage of IHD incident events or deaths under the base case scenario.

§ Health-adjusted life years and costs were discounted at 3%.

%E, per cent of total energy intake; IHD, ischemic heart disease; iTFA, industrially-derived trans-fatty acids; USD, United States dollar.

### Economic impact and cost-effectiveness

The reduced IHD burden was estimated to generate ~US$90 million (~₦12 billion) in total healthcare cost-savings in the first 10 years and ~US$520 million (~₦185 billion) over the population lifetime (table 2). The lifetime healthcare costs savings specific to IHD were estimated to be ~US$750 million (~₦270 billion). The greatest total healthcare cost-savings were attributed to men, among which 65% of the estimated population’s lifetime cost-savings accrued.

The implementation of the mandatory limit was estimated to cost the Nigerian government ~US$2.0 million (~₦720 million) in the first 10 years, accumulating to ~US$7.8 million (~₦2.8 billion) over the population lifetime (table 2 and online supplemental table S8). The cost for industry to reformulate foods containing iTFA was estimated to be ~US$15 million (~₦5.5 billion) in the first 10 years and ~US$18 million (~₦6.4 billion) over the population lifetime (table 2 and online supplemental table S8). Thus, the total implementation costs for the government and industry together were estimated to be ~US$17 million (~₦6.2 billion) in the first 10 years and ~US$26 million (~₦9.4 billion) over the population lifetime (table 2).

The implementation of a mandatory limit of iTFA content in Nigerian foods was estimated to be a net cost saving, with ~US$72 million (~₦26 billion) saved in the first 10 years, and ~US$490 million (~₦176 billion) over the population lifetime (table 2). The return of government investment (defined as healthcare cost-savings divided by government implementation costs) was estimated to US$46 (95% UI 39 to 53) per USD invested in the first 10 years, and US$66 (58 to 75) per USD invested over the population lifetime.

### Sensitivity analyses

**Figure 2:**
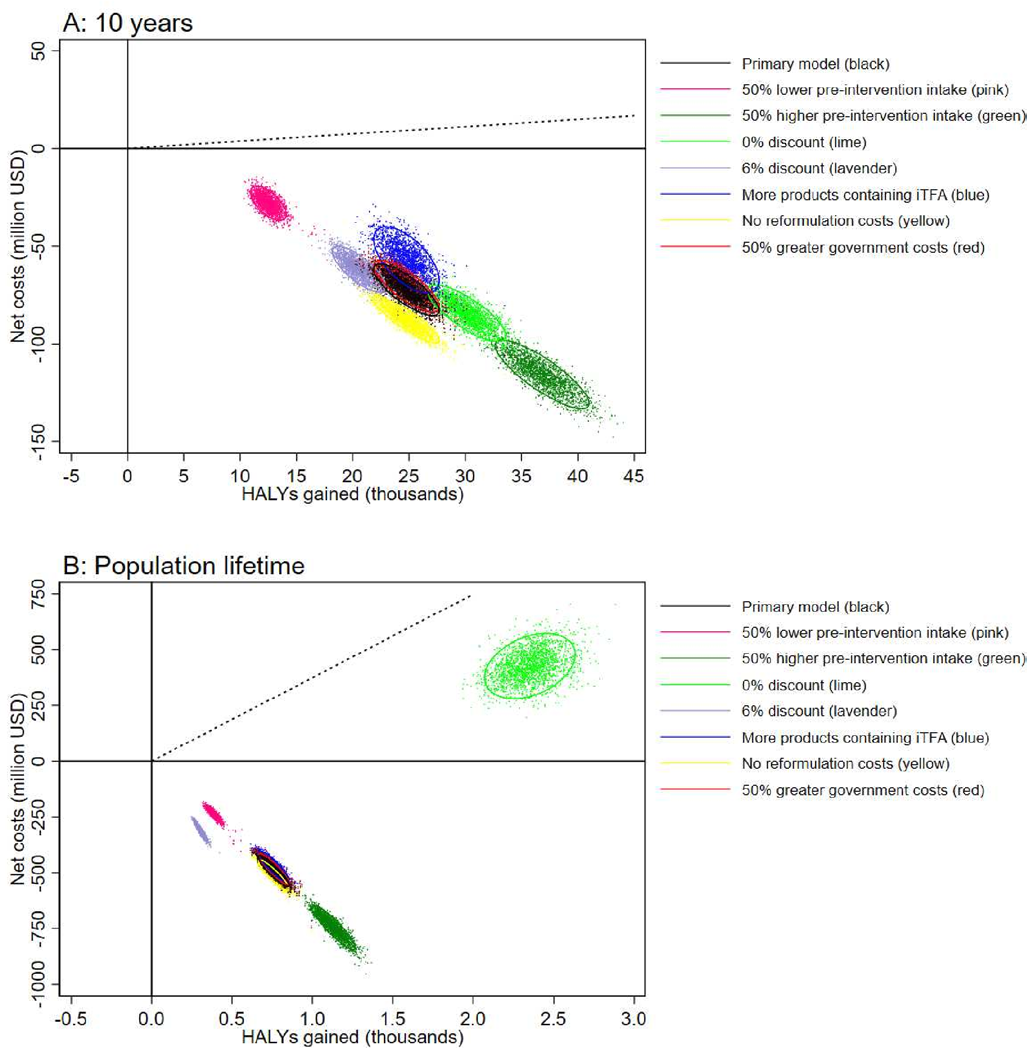
Net costs and health-adjusted life years (HALY) gained during the first 5 years (A) and over the population lifetime (B) estimated in the primary model and in deterministic sensitivity analyses related to preintervention trans-fatty acid intake, discount rate and implementation costs. The dotted line indicates the cost-effectiveness threshold for Nigeria (ie, US$374 per HALY gained). Figure created by the authors. iTFA, industrially derived trans-fatty acids.

The probability of the intervention to be cost-saving within the first 10 years was ≥99.9% in all sensitivity analyses (figure 2, online supplemental figure S1 and table S9), except when only a 0.05%E reduction in trans-fatty intake was assumed (probability of being cost saving: 43%; probability of being cost-effective: 64%). Also in the shorter time horizon (ie, 5 years), most sensitivity analyses suggested a high probability (≥70%) of being cost-saving (online supplemental figure S1), with the only exception being in scenarios with the smallest intake reduction (ie, ≤0.10%E). Over the population lifetime, all sensitivity analyses suggested the intervention had a 100% probability of being cost-saving, except when assuming a 0% discount rate. In this specific sensitivity analysis, with no discounting, the increased overall healthcare costs due to improved survival caused a positive net cost compared with the base population. Nevertheless, the intervention was estimated to have a 100% probability of being cost-effective (figure 2, online supplemental table S9). While assumptions regarding discount rates had the greatest impact over the population life time, assumptions regarding preintervention and postintervention intakes of *trans*-fatty acids and reformulation costs had the greatest impact on HALYs and/or net costs in the shorter time horizons (ie, ≤10 years). Alternative assumptions regarding policy implementation costs for the government had minimal impact on model estimates regardless of time horizon.

## Discussion

In this cost-effectiveness study, we estimated that a mandatory limit of iTFA (≤2% of total fat) in the Nigerian food supply would be a cost-saving intervention, compared with a scenario with no policy action to remove iTFA from foods. The intervention was estimated to prevent or postpone ~10 000 deaths and ~67 000 incident IHD events in the adult population over the first 10 years, and around ~260 000 IHD deaths and ~480 000 incident IHD events could be prevented over the population’s lifetime. The intervention was estimated to be cost-saving regardless of the time horizon evaluated.

Removing iTFA from the Nigerian food supply through a mandatory limit could lead to reductions in IHD burden and healthcare costs. The predicted costs for policy implementation (for both government and industry) were greatly outweighed by the estimated healthcare cost-savings already at 5 years after implementation. Even when considering total healthcare costs, which include additional costs for non-IHD related healthcare in a population where more individuals reach old age due to a lower IHD burden, the net cost-savings were substantial. The reduced spending on IHD care could potentially allow further action to help eliminate any remaining iTFA in the informal food sector (eg, subsidising iTFA-free oils and fats to street vendors). The WHO REPLACE package highlights the importance to support food manufacturers and vendors in the replacement of iTFA-containing fats.^6^ In our assessment of products with potentially iTFA-containing ingredients (eg, partially hydrogenated oils) in Nigerian supermarkets,^21^ most such products (218/310, 70%) came from outside of Nigeria (unpublished data). However, iTFA-containing ingredients may be used in Nigerian restaurant and street foods. Substantial amounts of elaidic acid (a major iTFA) have been reported in unbranded oils used in the preparation of suya, a popular Nigerian street food,^28^ and there are concerns that street foods could be a significant contributor to *trans*-fatty acid intake in low-income and middle-income countries.^29^ While prolonged or repeated heating of cooking oils at high temperatures may result in formation of trans-fats, common cooking practices rarely use such high temperatures.^30^ An alternative source of *trans*-fatty acids is the refinement process of cooking oils, especially deodorisation, which can generate substantial amounts of trans-fat isomers.^31^ Apart from intervention targeting iTFA, cost-savings on IHD care from the policy could also allow the allocation of funds to other preventive public health campaigns, thereby further increasing the potential health gains from the modelled iTFA policy.

Altering inputs and assumptions in a series of sensitivity analyses did not substantially change the findings suggesting that the intervention could be a cost-saving strategy to prevent IHD mortality and morbidity in Nigeria. Importantly, even if the policy would not result in the complete elimination of *trans*-fatty acid intake in Nigeria (eg, due to incomplete removal of iTFA from foods in Nigeria), the policy was still estimated to be a cost-saving or very cost-effective strategy to generate substantive health gains.

Interestingly, the intervention was estimated to be overall cost-saving, although the estimated mean *trans*-fatty acid intake before the intervention (ie, 0.25–0.31%E) is considerably lower compared with populations in previous cost-effectiveness analyses of policies targeting iTFA in Europe and Australia (ie, ≥0.59%E).^11 27 32^ Furthermore, even when healthcare cost-savings were disregarded, the intervention in Nigeria was still estimated to be cost-effective after 5 years.

A systematic review of policies aiming to lower *trans*-fatty acid intake suggested that bans of partially hydrogenated oils or mandatory iTFA limits (as modelled here) would outperform less stringent policies (eg, voluntary limits or mandatory labelling).^9^ This study expands this previous evidence by indicating that in Africa’s most populous nation, Nigeria, a lower-middle income country with low estimated *trans*-fatty acid intake, a mandatory limit of iTFA in foods, oils and fats could even generate net cost-savings. Such findings support national initiatives in Nigeria as well as WHO’s global call to eliminate iTFA from food supplies as a public health ‘best-buy’.^8^ With the REPLACE technical package, WHO provides guidance to countries for multiple strategies to be considered together in order to reduce or eliminate iTFA intake.^6^ For example, even if partially hydrogenated oils are banned, mandatory labelling of TFA content in foods is still recommended. Elimination of iTFA from the national food supply has proven feasible in high-income countries like Denmark and the USA,^33 34^ and an increasing number of middle-income countries have implemented WHO best-buy policies (eg, Brazil, India and Peru). In addition to government policy efforts to eliminate iTFA from foods, leading global food companies have pledged to remove iTFA from their products, which further supports the feasibility of iTFA elimination from foods.^35^ However, such voluntary actions by the food industry have often only led to a partial reduction of iTFA.^36^ Thus, government legislation to limit the use or completely ban iTFA-containing ingredients, with monitoring of compliance and enforcement mechanisms, will likely be needed to ensure elimination of iTFA from the food supply. By passing regulations to limit iTFA, Nigeria recently became the second country in Africa (after South Africa) with a best practice *trans-*fatty acid policy, as defined by WHO.^10^ The findings of our study may aid the implementation of the regulation in Nigeria and, perhaps more importantly, they can provide guidance and motivation to policy-makers considering similar policies for low-income and middle-income countries in Africa and globally.

We recently conducted a similar cost-effectiveness analysis of a mandatory iTFA limit in Kenya.^12^ Due to substantial dissimilarities in data inputs (eg, demographics and cost inputs), the magnitude of estimated health and economic impacts varied considerably between the studies. Nevertheless, a mandatory iTFA limit in foods, fats and oils was estimated to generate substantial net cost-savings while markedly reducing the IHD burden in both countries. In Kenya, the probability of the policy being cost-saving was ≥99.6% across all sensitivity analyses and regardless of time horizon.

Our study has several strengths. To identify products potentially containing iTFA in the Nigerian food supply, we used a country-specific and contemporary database of packaged foods in Nigeria.^21^ We derived the relative risk estimates of *trans*-fatty acid intake with incident IHD from a large meta-analysis of prospective studies directly linking consumption of *trans*-fatty acid to incidence of IHD, thereby taking into consideration not only effects mediated by blood lipids but also other potential pathways through which *trans*-fatty acid intake can impact IHD, for example, inflammation.^37^ Total healthcare costs included both costs related to IHD care and other healthcare, thereby accounting for potential additional costs resulting from a population where more individuals reach old age due to a reduced IHD burden. We estimated health impact and healthcare cost-savings stratified by sex.

We acknowledge that our study has limitations. In the absence of nationally representative estimates of *trans-*fatty acid intake in Nigeria, we used age-specific and sex-specific intake estimates from the GBD 2019 study derived through imputation using a spatiotemporal Gaussian regression method, which uses dietary data and PHVO sales as inputs.^38^ We did not consider any potential ongoing voluntary reformulation to reduce iTFA in Nigeria’s food supply, as there is little evidence to suggest such secular trends. For example, trans-fatty acid intake estimates from GBD and hydrogenated vegetable oil sales data from Euromonitor show no meaningful changes in recent years.^1 39^ In addition, even in a sensitivity analysis assuming 50% lower baseline intake (compared with the main analysis), the policy was estimated to be cost-saving. Thus, it is very likely that the policy would also be cost-saving even with some ongoing trends of reduced iTFA intake due to voluntary reformulation. We used estimates of industry costs from the UK, reported nearly 20 years ago, and may thus not be fully representative of the potential reformulation costs required to comply with the modelled policy. Furthermore, our estimation of potentially iTFA-containing products requiring reformulation did not include foods or ingredients not available in supermarkets. However, even when assuming doubled reformulation costs in a sensitivity analysis, the policy was likely to be cost-saving or at least very cost-effective already after 5 years. In the absence of a recent costs-of-illness study for Nigeria, we used IHD costs from a rural area with rather uncertain estimates (likely lower compared with urban areas), assuming a triangular distribution. Thus, the estimates may not fully represent national IHD costs, but are more likely underestimated than overestimated. Thus, we may underestimate the healthcare cost-savings of our preventive intervention, while overestimating the healthcare costs in added years of life, as these are calculated by subtracting the IHD costs from overall healthcare expenditure. We are thus erring on the side of caution; higher estimates of IHD care costs would not alter the conclusion that an iTFA limit is highly likely to be cost-saving. We evaluated the cost-effectiveness from an extended healthcare perspective, including healthcare costs both IHD-specific and non-IHD costs, as well as policy implementation costs for the government and the food industry. Thus, the societal savings from the intervention are likely to be substantially underestimated, given that we did not consider indirect costs (eg, productivity loss due to absenteeism or disability). Due to the scarceness of data (eg, on *trans*-fatty acid intake or IHD burden), we were able to stratify our analyses on sex, but not on socioeconomic status or urban versus rural residency. Previous analyses from the UK and Australia have indicated that elimination of iTFA from the food supply could reduce social-economic and urban–rural inequalities in IHD disease burden.^11 27^ The potential impact of such inequalities in Nigeria is unclear. Like previous cost-effectiveness analyses of iTFA-targeting policies, we used risk estimates of change in trans-fatty acid against the overall diet rather than specific substitution with other fat classes. If iTFAs were systematically replaced by saturated fatty acids, the impact of the mandatory limit could be lower than estimated here. However, a systematic review of iTFA policies suggests no overall increase in saturated fatty acid content among foods reduced in iTFA content.^40^

### Conclusion

Compared with a base scenario with sustained intake of *trans*-fatty acid at current levels, a mandatory limit of iTFAs was estimated to be a cost-saving strategy to avert hundreds of thousands of IHD events and premature deaths in Nigeria. Our findings support the recently passed regulation to reduce iTFA content in the Nigerian food supply.

## Supplementary material

10.1136/bmjgh-2023-014294 online supplemental file 1

10.1136/bmjgh-2023-014294 online supplemental file 2

## Data Availability

Data are available upon reasonable request. All data relevant to the study are included in the article or uploaded as supplementary information.
