# Supplementary material for: Estimated health benefits, costs and cost-effectiveness of eliminating industrial trans-fatty acids in Nigeria: cost-effectiveness analysis
Source: BMJ Glob Health. 2024 Apr 17;9(4):e014294. doi: 10.1136/bmjgh-2023-014294 (PMC11029410; doi:10.1136/bmjgh-2023-014294)
Supplement: online supplemental file 1 [file bmjgh-9-4-s001.pdf]

Online supplemental material

Appendix

Study proposal and analytical plan

Title: Estimated health benefits, costs, and cost-effectiveness of policies to reduce the intake of industrial trans-fatty acids in Nigeria

Background: A high intake of trans fatty acids (TFAs) is a well-known dietary risk factor of ischemic heart disease (IHD). TFA causes cardiometabolic dysfunction,[1] and a meta-analysis of prospective studies reported that for every 2% of total energy from TFAs, IHD risk increased by 23%.[2] TFAs occur naturally at low levels in meat and milk from ruminants, but in most countries intakes are predominantly driven by the use of partially hydrogenated vegetable oils in a variety of foods such as packaged processed products and street foods. [3]

Given the adverse effects of TFAs, the World Health Organization (WHO) recommends limiting TFA intake to <1 percent of energy (%E) and countries have implemented strategies to reduce industrially derived TFA (iTFA) in the food supply[4,5]. These policies range from voluntary reformulation, mandatory labeling, through to banning partially hydrogenated vegetable oils entirely. For example, iTFA content in foods must be ≤2% of total fat in Denmark[6] and in 2015, the US Food and Drug Administration determined that partially hydrogenated oils are no longer considered generally recognized as safe for use in human food, essentially eliminating its use.[7] However, in many other countries policies to reduce iTFA have been limited or are completely lacking.

While removal of iTFA from the food supply is expected to have the greatest impact in places where intake is high, we recently estimated that a ban of iTFA even in Australia (a country with relatively low iTFA) would be cost-saving or highly cost-effective and could likely help reduce health disparities. [8] Similar cost-effectiveness analyses for low- and middle-income countries (LMIC) are scarce, although the findings could help inform policy makers in resource-limited settings on the overall health and economic impact of removing iTFA from the food supply.

The 2019 Nigerian National Multisectoral Action Plan for the Prevention and Control of Noncommunicable Diseases includes a high-level political commitment to eliminate artificial trans fatty acids (TFA) through

reformulation, fiscal policies, food labelling changes, and agricultural policies. In 2020, the Nigerian Agency for Food and Drug Administration and Control (NAFDAC) created a draft policy to limit TFA to <2% of fat content in all food products, which aligns with the WHO REPLACE package. However, the potential health impact, costs and the cost-effectiveness of TFA elimination in Nigeria are unknown.

Aims and Objectives: To estimate the health impact, costs, and cost-effectiveness of a mandatory TFA limit in the Nigerian food supply. The specific aims include to:

1. Develop a Markov-cohort model framework to estimate the IHD burden in Nigeria for:
    a. a base case scenario under current TFA intake
    b. alternative scenarios with policies to reduce TFA intake
2. Estimate the direct healthcare cost (IHD-related and total) under both scenarios.
3. Estimate the implementation cost of each policy.
4. Estimate cost-effectiveness, compared to the base case scenario, of the policies, based on net costs and potential health gains.

Expected outcomes: The findings from this study may help to motivate policy makers and the public health community to further commit to and invest into policies related to reducing TFA intake for heart disease prevention. The expected outcomes include:

- Averted IHD deaths, number of IHD events, health-adjusted life-years (HALYs), and healthcare savings (total and IHD-related) from reduced TFA intake.
- Policy costs (could include e.g., legislation, monitoring, and reformulation costs).
- Incremental cost-effectiveness ratio (ICER).

Policy scenario: A limit of iTFA content (≤2%E of all fats) in the Nigerian food supply. The limit would target both packaged foods and foods purchased at restaurants or from street vendors

Model and analysis: A multiple cohort proportional multistate life table (Markov) framework will be developed to estimate the potential impact of reduced iTFA intake on IHD mortality and morbidity as well as HALYs and direct healthcare (IHD-related and total) costs (**Figure 1**). We will incorporate data on TFA intake, IHD burden (e.g., incidence, prevalence, case fatality, disability weights), and all-cause mortality, from country- or region-specific sources or the Global Burden of Disease study (**Table 1**). The effects of TFA intake on IHD risk will be derived from previous meta-analyses of observational studies. Direct IHD-related and total healthcare costs including hospital services, out-of-hospital medical services, pharmaceuticals, and health professionals will be based on best available evidence from national or global data sources and literature. Cost-effectiveness will be evaluated from an extended healthcare perspective (incorporating policy and direct healthcare costs) over 10 years and over the population lifetime.[7] The incremental cost-effectiveness ratio (ICER) will be calculated by dividing the incremental net cost (i.e., policy costs minus healthcare cost savings) by the incremental HALYs gained. Cost-saving will be defined as having a negative incremental net cost and we will use WHO benchmarks and country-specific willingness to pay thresholds for defining cost-effectiveness. We will present estimated health gains separately and jointly for men and women. The parameter uncertainty around the modelled estimates will be quantified using Monte Carlo simulations, with random draws made for each iteration from the distributions of model inputs (e.g., TFA intake, policy effect, relative risks, and costs). Deterministic sensitivity analyses will be conducted to explore the impact of variations in data inputs and model assumption (e.g., counterfactual TFA intake, policy implementation costs, and discount rate).

Health outcomes

We used data from the GBD 2019 study to estimate baseline incidence, prevalence, and mortality rates for IHD in Nigeria.[9] Using multiple data sources, the GBD study provides a systematic analysis of the burden of disease in 204 countries and territories. A complete list of data sources is available through the GBD 2019 data input sources.[10] Furthermore, we used the DisMod II software[11] to obtain estimates of case fatality rates for IHD, as these are seldom reported in the literature. Using data on incidence, prevalence, mortality, and population numbers for Nigeria, DisMod II generates estimates of IHD case fatality rates. The software uses

a set of differential equations that leverage the causal relation in a typical disease process to estimate absent epidemiological parameters while maintaining stability in the overall disease epidemiology.[11] These analyses were conducted by sex and five-year age groups.

Disability weights (DW) indicate the average loss of quality of life due to a disease condition.[12] We calculated DW by dividing the number of IHD-specific years lived with disability (YLD) by the number of people living with IHD for each age/sex group. These DWs were adjusted for the disability due to other health conditions in each age group, given that the starting point for calculating YLDs was less than full health. We inflated by '1-pYLD /n', whereby pYLD stands for the total YLD for all diseases except IHD, and n is the population size. This calculation is done to derive DWs at the cause level (e.g., IHD) instead of sequela level (e.g., myocardial infarction (MI), chronic IHD, angina, asymptomatic IHD following MI, acute MI) as reported in the GBD study.[9] We base our calculations on the GBD methods for estimating YLDs as the disease prevalence multiplied by disability weights.[12] These DWs are used in the calculation of health-adjusted life years.

Healthcare costs

A review of the literature was conducted to determine total health expenditure and the healthcare costs associated with IHD in Nigeria. Our model includes costs of incident IHD and costs for prevalent IHD. We used estimates from a study by Rosendaal et al.,[13] that included data from a micro-costing study at a public primary care facility[14] on the costs of hypertension and cardiovascular disease prevention care according to international guidelines. This was further informed by additional data on resource utilization from the University of Ilorin Teaching Hospital.[13] Using a healthcare provider perspective, the authors estimated the costs of acute care after an IHD event per patient (i.e., incident IHD costs) and costs of follow-up care after an IHD event per patient per year (i.e., prevalent IHD costs). The cost estimates included consultation fees, diagnostic tests, hospital bed-days, medication as well as overheads. These IHD costs were estimated in 2012 USD, which we inflated to 2019 values using World Bank consumer price indices for Nigeria .[15]

Estimates of total health expenditure were obtained from the Nigerian National Health Accounts for 2017 report.[16] Due to data limitations for Nigeria, we used data from the Kenyan 2013 Household Health Expenditure and Utilization Survey[17] to derive age and sex distributions of total health expenditure in Nigeria. The total health expenditure was then divided by population numbers in each sex and age-group to derive the

per capita expenditure. Our model accounts for healthcare costs that may arise from other conditions that result from keeping people alive due to the intervention. To estimate these unrelated/ non-IHD costs, we subtract the IHD costs from the total health expenditure for each sex and age-group. Total health expenditure estimates were inflated from 2017 to 2019 (model base year) using World Bank consumer price indices for Nigeria.[15]

Estimating government costs for policy implementation

Government costs for policy implementation were elicited by a group of experts who are tenured staff of National Agency for Food Drug and Administration Control (NAFDAC) which comprised a Food Safety Specialist and Risk Analyst, two Economists with one being a Chartered Accountant and two Biostaticians. The team collected data from the Federal Executive and National Assembly as these bodies were identified to be involved in public health policy formulation, laws enactment, standards elaboration and regulations development. Data was also collected from the Ministries of Health, Industries, Trade and Investment, and from National Agency for Food and Drug Administration and Control NAFDAC, Standards Organisation of Nigeria SON involved in food safety, public health. The sources of data were from the libraries and websites for annual work plans, work activities, and the outcomes of these agencies and departments. Other sources of data were from planned budgets and records of expenditure and procurement from these agencies and departments. Responsible officers were also engaged to draw a correlation between the sourced figures and their pronouncements.

Estimating industry costs for policy implementation

As previously described, we identified 310 food products potentially containing iTFA in the Nigeria food supply.[18] Of these 310 products, n=64 (20.6%) were manufactured in Nigeria, n=218 (70.3%) were produced abroad, and n=28 (9.0%) did not contain information of country of origin. Due to paucity of reliable Nigerian data on industry reformulation costs, our calculations were based on evidence from the United Kingdom.[19] We estimated initial reformulation costs (i.e., additional costs to reformulate and repackage, including loss through disuse of existing packaging, products outside of natural product cycles) in the first year by calculating the corresponding USD costs from the UK estimates (£25,000 per product) multiplied by the number of products in the Nigerian food supply potentially containing iTFA. Furthermore, we assumed an ongoing

annual industry cost equivalent to 1% of the initial reformulation cost to conservatively account for increased industry costs for using alternative ingredients, which is consistent with previous studies.[19,20]

All government and industry costs were inflated to 2019 values using the World Bank consumer price indices for Nigeria.[15]

Estimation of policy effect on disease incidence

To estimate the proportional change in IHD incidence due to the elimination of iTFAs, we used the reference and intervention *trans*-fatty acid intakes and the relative risk of IHD per %E of *trans*-fatty acid intake to calculate the potential impact fraction, according to Barendregt's continuous 'distribution shift' potential impact fraction method (Annexure 2). [21]

$$\mathrm{PIF}_{as} = \frac{\int_{x=0}^{m} RR_a(x)P_{as}(x)dx - \int_{x=0}^{m} RR_a(x)P'_{as}(x)dx}{\int_{x=0}^{m} RR_a(x)P_{as}(x)dx} \qquad \text{Eq. 1}$$

Here, the PIF_{as} is the potential impact fraction for age group a and sex s, $RR_a(x)$ is the relative risk for age group a as a function of the exposure x (i.e., *trans*-fatty acid intake), $P_{as}(x)$ is the reference *trans*-fatty acid intake distribution of age group a and sex s, and $P'_{as}(x)$ is the intervention *trans*-fatty acid intake distribution for the same age-sex group. For the primary model, where we assumed that the policy would virtually eliminate intake of *trans*-fatty acids, a simplification of the PIF equation (i.e., a population attributable fraction equation) was used (Equation 2).

$$\mathrm{PIF}_{as} = \frac{\int_{x=0}^{m} RR_a(x)P_{as}(x)dx - 1}{\int_{x=0}^{m} RR_a(x)P_{as}(x)dx} \qquad \text{Eq. 2}$$

The PIF was used to calculate the effect on IHD incidence due to the reduction in *trans*-fatty acid intake (Equation 3).

$$I' = I(1 - PIF) \qquad \text{Eq. 3}$$

Here, I is the IHD incidence in the reference population, I' is the IHD incidence in the intervention population, and PIF is the potential impact fraction.

Table S1. Baseline (pre-policy) in intake of total TFA by age and sex.[1]

		Pre-policy intake (%E)	
**Sex**	**Age (years)**	**Mean**	**SD**
Women	20-24	n/a[2]	n/a[2]
	25-29	0.25 (0.17; 0.35)	0.14 (0.09; 0.20)
	30-34	0.25 (0.17; 0.36)	0.14 (0.09; 0.20)
	35-39	0.25 (0.18; 0.35)	0.14 (0.09; 0.19)
	40-44	0.26 (0.17; 0.36)	0.14 (0.09; 0.20)
	45-49	0.26 (0.18; 0.37)	0.14 (0.10; 0.20)
	50-54	0.27 (0.19; 0.37)	0.14 (0.10; 0.20)
	55-59	0.27 (0.19; 0.39)	0.15 (0.10; 0.22)
	60-64	0.28 (0.20; 0.39)	0.15 (0.11; 0.21)
	65-69	0.28 (0.20; 0.40)	0.15 (0.11; 0.22)
	70-74	0.29 (0.20; 0.40)	0.16 (0.11; 0.22)
	75-79	0.30 (0.21; 0.42)	0.16 (0.11; 0.23)
	80-84	0.31 (0.21; 0.43)	0.17 (0.11; 0.24)
	85-89	0.30 (0.21; 0.41)	0.17 (0.11; 0.23)
	90-94	0.31 (0.21; 0.42)	0.17 (0.11; 0.24)
	95+	0.31 (0.21; 0.43)	0.17 (0.11; 0.24)
Men	20-24	n/a[2]	n/a[2]
	25-29	0.25 (0.17; 0.35)	0.14 (0.09; 0.19)
	30-34	0.25 (0.17; 0.36)	0.14 (0.09; 0.20)
	35-39	0.26 (0.18; 0.35)	0.14 (0.10; 0.19)
	40-44	0.26 (0.18; 0.36)	0.14 (0.10; 0.20)
	45-49	0.26 (0.18; 0.37)	0.14 (0.10; 0.20)
	50-54	0.27 (0.19; 0.37)	0.14 (0.10; 0.20)
	55-59	0.27 (0.19; 0.38)	0.15 (0.10; 0.21)
	60-64	0.28 (0.19; 0.38)	0.15 (0.10; 0.21)
	65-69	0.28 (0.19; 0.39)	0.16 (0.10; 0.22)
	70-74	0.29 (0.20; 0.41)	0.16 (0.11; 0.23)
	75-79	0.30 (0.21; 0.42)	0.16 (0.11; 0.23)
	80-84	0.31 (0.21; 0.43)	0.17 (0.11; 0.24)
	85-89	0.31 (0.21; 0.43)	0.17 (0.11; 0.24)
	90-94	0.31 (0.22; 0.43)	0.17 (0.12; 0.24)
	95+	0.30 (0.21; 0.42)	0.17 (0.11; 0.23)

%E, percentage of energy intake; n/a, SD, standard deviation.

[1]in the absence of national data on trans-fatty acid intake in Nigeria, we utilized estimates from the Global Burden of Disease study,[22] provided by the Institute for Health Metrics and Evaluation (IHME). A spatio-temporal Gaussian process regression framework using data from 24-h recalls, food frequency questionnaires, and sales data of partially hydrogenated vegetable oils from Euromonitor, were used to generate country-, year-, age- and sex-specific estimates of trans-fatty acids

[2]Estimates for ages 20-24 years not available, thus estimates for ages 25-29 years used for both age groups.

Table S2. Population size and mortality rate, by sex and age.

	Population size (1000s)		Mortality rate (per 1000)	
**Age**	**Women**	**Men**	**Women**	**Men**
20	2235.0	1989.1	1.2	1.5
21	2161.9	1903.9	1.2	1.5
22	2092.5	1824.5	1.2	1.5
23	2027.8	1751.4	1.2	1.5
24	1955.0	1676.4	1.2	1.5
25	1873.6	1598.1	1.7	2.0
26	1794.6	1528.5	1.7	2.0
27	1717.8	1466.5	1.7	2.0
28	1644.4	1412.5	1.7	2.0
29	1577.0	1362.2	1.7	2.0
30	1512.2	1313.5	2.5	2.7
31	1455.2	1267.0	2.5	2.7
32	1405.5	1224.1	2.5	2.7
33	1361.5	1184.9	2.5	2.7
34	1311.7	1146.1	2.5	2.7
35	1256.0	1107.6	3.3	3.9
36	1205.2	1072.2	3.3	3.9
37	1159.3	1039.8	3.3	3.9
38	1117.7	1010.0	3.3	3.9
39	1076.3	980.2	3.3	3.9
40	1035.1	950.1	4.3	5.5
41	996.1	919.4	4.3	5.5
42	960.2	888.9	4.3	5.5
43	927.4	858.8	4.3	5.5
44	895.2	829.8	4.3	5.5
45	864.3	802.2	5.7	7.3
46	835.7	772.7	5.7	7.3
47	809.7	741.5	5.7	7.3
48	786.6	708.5	5.7	7.3
49	763.4	674.9	5.7	7.3
50	739.6	640.1	7.8	10.1
51	709.5	605.2	7.8	10.1
52	675.7	571.7	7.8	10.1
53	639.5	539.2	7.8	10.1
54	610.1	504.6	7.8	10.1
55	587.5	469.3	10.9	14.2
56	563.2	439.7	10.9	14.2
57	534.9	413.1	10.9	14.2
58	503.3	389.2	10.9	14.2
59	472.2	365.2	10.9	14.2
60	441.8	341.5	16.9	21.4
61	408.8	319.2	16.9	21.4
62	374.3	298.4	16.9	21.4
63	339.5	278.8	16.9	21.4
64	308.2	258.5	16.9	21.4
65	280.1	238.1	25.6	31.6
66	257.8	221.8	25.6	31.6
67	240.1	208.9	25.6	31.6
68	222.1	198.7	25.6	31.6
69	246.6	210.2	25.6	31.6
70	163.1	162.8	43.1	49.2
71	159.6	159.8	43.1	49.2
72	148.8	151.4	43.1	49.2
73	121.9	134.5	43.1	49.2
74	109.9	122.8	43.1	49.2
75	144.7	133.0	67.5	74.2
76	131.8	122.4	67.5	74.2
77	118.9	110.9	67.5	74.2
78	93.0	94.9	67.5	74.2
79	86.0	87.4	67.5	74.2

80	75.8	77.7	103.7	111.6
81	65.9	68.6	103.7	111.6
82	56.6	60.0	103.7	111.6
83	47.8	50.9	103.7	111.6
84	40.9	44.0	103.7	111.6
85	34.9	37.5	140.1	157.9
86	29.9	31.6	140.1	157.9
87	25.5	26.4	140.1	157.9
88	19.8	23.3	140.1	157.9
89	16.8	18.7	140.1	157.9
90	14.1	14.6	199.8	227.5
91	11.6	11.2	199.8	227.5
92	9.5	8.4	199.8	227.5
93	8.7	5.6	199.8	227.5
94	6.8	4.0	199.8	227.5
95	3.6	2.5	303.8	334.9
96	3.0	1.8	303.8	334.9
97	2.5	1.3	303.8	334.9
98	2.1	0.9	303.8	334.9
99	1.7	0.7	303.8	334.9
100+	5.1	1.4	-[1]	-[1]

[1]Model simulations terminated when all individuals were dead or had reached the age 100 years.

Table S3. Estimated rates of IHD incidence and prevalence (per 100K) and IHD case fatality by age and sex.

	Women			Males		
**Age**	**Incidence (per 100K)**	**Prevalence (per 100K)**	**Case fatality (%)**	**Incidence (per 100K)**	**Prevalence (per 100K)**	**Case fatality (%)**
20	0	20	3.6	10	30	4.0
21	0	20	3.3	10	30	3.8
22	10	30	3.0	10	40	3.6
23	10	30	2.6	10	50	3.3
24	10	40	2.2	10	60	3.0
25	10	40	1.9	20	80	2.7
26	10	50	1.6	20	90	2.4
27	10	70	1.3	20	110	2.2
28	20	80	1.2	30	140	2.0
29	20	90	1.1	30	160	2.0
30	20	110	1.2	40	190	2.0
31	20	130	1.2	40	230	2.0
32	20	150	1.3	40	260	2.1
33	30	180	1.4	50	300	2.1
34	30	200	1.4	50	350	2.2
35	30	230	1.4	60	390	2.2
36	30	260	1.4	60	440	2.3
37	40	290	1.4	70	490	2.3
38	40	320	1.5	70	550	2.4
39	50	360	1.5	80	610	2.4
40	50	400	1.5	90	680	2.4
41	60	450	1.6	100	750	2.5
42	60	500	1.7	110	830	2.5
43	70	560	1.7	120	920	2.5
44	80	620	1.8	140	1,030	2.6
45	90	690	1.9	150	1,140	2.6
46	100	770	2.1	170	1,270	2.6
47	110	860	2.2	190	1,410	2.7
48	120	950	2.4	210	1,570	2.7
49	140	1,050	2.5	230	1,740	2.8
50	150	1,170	2.6	260	1,930	2.8
51	170	1,290	2.7	290	2,140	2.8
52	180	1,430	2.8	320	2,370	2.9
53	200	1,570	2.9	350	2,630	2.9
54	220	1,740	2.9	390	2,910	2.9
55	250	1,920	3.0	430	3,220	2.9
56	270	2,110	3.0	470	3,560	2.9
57	300	2,330	3.1	520	3,940	2.9
58	340	2,570	3.3	580	4,350	3.0
59	370	2,820	3.4	640	4,800	3.0
60	410	3,100	3.6	700	5,290	3.1
61	450	3,400	3.8	770	5,820	3.1
62	500	3,730	4.0	840	6,390	3.2
63	540	4,070	4.2	910	7,010	3.3
64	590	4,430	4.4	990	7,660	3.3
65	640	4,820	4.7	1,060	8,360	3.4
66	690	5,220	4.9	1,140	9,090	3.5
67	750	5,640	5.2	1,230	9,860	3.5
68	810	6,080	5.4	1,320	10,680	3.6
69	880	6,540	5.7	1,410	11,530	3.7
70	950	7,010	6.0	1,520	12,430	3.8
71	1,020	7,510	6.3	1,620	13,360	3.9
72	1,100	8,020	6.7	1,730	14,330	4.0
73	1,190	8,560	7.0	1,860	15,350	4.1
74	1,290	9,100	7.4	1,980	16,400	4.3
75	1,380	9,660	7.8	2,120	17,490	4.4
76	1,480	10,240	8.2	2,250	18,620	4.6
77	1,590	10,810	8.6	2,400	19,780	4.7
78	1,690	11,400	9.1	2,550	20,970	4.9
79	1,800	11,970	9.6	2,700	22,180	5.1
80	1,910	12,540	10.1	2,840	23,410	5.3
81	2,020	13,090	10.7	2,990	24,640	5.5
82	2,120	13,610	11.3	3,120	25,860	5.7
83	2,220	14,090	12.0	3,260	27,070	6.0
84	2,320	14,520	12.7	3,390	28,240	6.3
85	2,420	14,900	13.5	3,510	29,360	6.7
86	2,510	15,200	14.5	3,610	30,400	7.2
87	2,600	15,420	15.5	3,700	31,340	7.7
88	2,690	15,550	16.7	3,790	32,150	8.4
89	2,780	15,580	18.0	3,860	32,810	9.1
90	2,860	15,510	19.4	3,920	33,300	9.9
91	2,930	15,340	20.9	3,970	33,620	10.9
92	3,010	15,070	22.4	4,020	33,750	11.8
93	3,070	14,730	23.9	4,070	33,700	12.7

94	3,130	14,330	25.4	4,120	33,500	13.6
95	3,190	13,890	26.9	4,170	33,150	14.5
96	3,240	13,410	28.4	4,220	32,670	15.4
97	3,280	12,930	29.7	4,260	32,080	16.2
98	3,320	12,450	31.1	4,310	31,410	17.0
99	3,360	11,990	32.4	4,350	30,670	17.8
100+	3,380	11,570	32.9	4,370	29,910	18.1

Table S4. Age- and sex-specific disability weights for IHD, adjusted for disability from other causes.

**Disease**	**Ischemic heart disease**	
**Age/ Sex**	**Male**	**Female**
**20 to 24**	0.022	0.021
**25 to 29**	0.024	0.021
**30 to 34**	0.024	0.020
**35 to 39**	0.025	0.021
**40 to 44**	0.029	0.023
**45 to 49**	0.030	0.024
**50 to 54**	0.029	0.023
**55 to 59**	0.029	0.023
**60 to 64**	0.029	0.023
**65 to 69**	0.029	0.022
**70 to 74**	0.030	0.023
**75 to 79**	0.030	0.024
**80 to 84**	0.030	0.023
**85 to 89**	0.028	0.021
**90 to 94**	0.029	0.021
**95 plus**	0.033	0.027

Table S5. All-cause prevalent years lived with disability (pYLD) rates by sex and single-year age groups.

Age	Male	Female	Age	Male	Female	Age	Male	Female
**20**	0.07	0.09	**50**	0.13	0.16	**80**	0.28	0.29
**21**	0.07	0.09	**51**	0.13	0.16	**81**	0.28	0.29
**22**	0.07	0.09	**52**	0.13	0.16	**82**	0.29	0.30
**23**	0.07	0.09	**53**	0.14	0.17	**83**	0.29	0.30
**24**	0.07	0.10	**54**	0.14	0.17	**84**	0.30	0.31
**25**	0.08	0.10	**55**	0.14	0.17	**85**	0.30	0.31
**26**	0.08	0.10	**56**	0.15	0.17	**86**	0.31	0.31
**27**	0.08	0.11	**57**	0.15	0.18	**87**	0.31	0.32
**28**	0.08	0.11	**58**	0.15	0.18	**88**	0.32	0.32
**29**	0.08	0.11	**59**	0.16	0.18	**89**	0.32	0.33
**30**	0.09	0.12	**60**	0.16	0.19	**90**	0.33	0.33
**31**	0.09	0.12	**61**	0.17	0.19	**91**	0.33	0.33
**32**	0.09	0.12	**62**	0.17	0.19	**92**	0.33	0.34
**33**	0.09	0.13	**63**	0.18	0.20	**93**	0.34	0.34
**34**	0.09	0.13	**64**	0.18	0.20	**94**	0.35	0.35
**35**	0.09	0.13	**65**	0.19	0.21	**95**	0.35	0.36
**36**	0.10	0.13	**66**	0.19	0.21	**96**	0.36	0.36
**37**	0.10	0.14	**67**	0.20	0.22	**97**	0.37	0.37
**38**	0.10	0.14	**68**	0.20	0.22	**98**	0.37	0.38
**39**	0.10	0.14	**69**	0.21	0.23	**99**	0.38	0.38
**40**	0.10	0.15	**70**	0.22	0.24	**100**	0.38	0.38
**41**	0.11	0.15	**71**	0.22	0.24			
**42**	0.11	0.15	**72**	0.23	0.25			
**43**	0.11	0.15	**73**	0.24	0.25			
**44**	0.11	0.15	**74**	0.24	0.26			
**45**	0.12	0.15	**75**	0.25	0.26			
**46**	0.12	0.16	**76**	0.25	0.27			
**47**	0.12	0.16	**77**	0.26	0.27			
**48**	0.12	0.16	**78**	0.27	0.28			
**49**	0.12	0.16	**79**	0.27	0.28			

Years lived with disability (YLDs) and Nigeria population data estimates from the 2019 GBD study used to calculate per person YLD [10].

Table S6. Total, IHD-related, and other healthcare costs per capita, by sex and age.

	Annual pre-intervention healthcare costs per capita, USD					
	Women			Males		
Age (year)	Total	IHD	Non-IHD	Total	IHD	Non-IHD
20	24.8	0.1	24.8	19.8	0.1	19.7
21	24.8	0.1	24.8	19.8	0.1	19.7
22	24.9	0.1	24.8	19.8	0.1	19.7
23	24.9	0.1	24.8	19.9	0.2	19.7
24	24.9	0.1	24.8	19.9	0.2	19.7
25	39.3	0.1	39.2	32.3	0.3	32.0
26	39.3	0.2	39.2	32.3	0.3	32.0
27	39.4	0.2	39.2	32.4	0.4	32.0
28	39.4	0.3	39.2	32.5	0.5	32.0
29	39.5	0.3	39.2	32.5	0.6	32.0
30	39.5	0.4	39.2	32.7	0.7	32.0
31	39.6	0.4	39.2	32.8	0.8	32.0
32	39.7	0.5	39.2	32.9	0.9	32.0
33	39.8	0.6	39.2	33.0	1.0	32.0
34	39.8	0.7	39.2	33.2	1.2	32.0
35	76.1	0.8	75.3	59.1	1.3	57.8
36	76.2	0.9	75.3	59.3	1.5	57.8
37	76.3	1.0	75.3	59.5	1.7	57.8
38	76.4	1.1	75.3	59.7	1.8	57.8
39	76.5	1.2	75.3	59.9	2.1	57.8
40	76.7	1.3	75.3	60.1	2.3	57.8
41	76.8	1.5	75.3	60.3	2.5	57.8
42	77.0	1.7	75.3	60.6	2.8	57.8
43	77.2	1.9	75.3	60.9	3.1	57.8
44	77.4	2.1	75.3	61.3	3.5	57.8
45	166.4	2.3	164.1	132.6	3.8	128.8
46	166.7	2.6	164.1	133.1	4.3	128.8
47	167.0	2.9	164.1	133.5	4.8	128.8
48	167.3	3.2	164.1	134.1	5.3	128.8
49	167.7	3.6	164.1	134.6	5.9	128.8
50	168.0	3.9	164.1	135.3	6.5	128.8
51	168.5	4.4	164.1	136.0	7.2	128.8
52	168.9	4.8	164.1	136.8	8.0	128.8
53	169.4	5.3	164.1	137.7	8.9	128.8
54	170.0	5.9	164.1	138.6	9.8	128.8
55	421.0	6.5	414.6	376.6	10.9	365.7
56	421.7	7.1	414.6	377.7	12.0	365.7
57	422.4	7.9	414.6	379.0	13.3	365.7
58	423.3	8.7	414.6	380.4	14.7	365.7
59	424.1	9.5	414.6	381.9	16.2	365.7
60	425.0	10.5	414.6	383.5	17.8	365.7
61	426.1	11.5	414.6	385.3	19.6	365.7
62	427.2	12.6	414.6	387.2	21.5	365.7
63	428.3	13.8	414.6	389.3	23.6	365.7
64	429.6	15.0	414.6	391.5	25.8	365.7
65	903.0	16.3	886.7	668.5	28.1	640.4
66	904.3	17.7	886.7	671.0	30.5	640.4
67	905.7	19.1	886.7	673.5	33.1	640.4
68	907.2	20.6	886.7	676.3	35.8	640.4
69	908.8	22.1	886.7	679.1	38.6	640.4
70	910.4	23.8	886.7	682.1	41.6	640.4
71	912.1	25.5	886.7	685.2	44.7	640.4
72	913.9	27.2	886.7	688.4	48.0	640.4
73	915.7	29.1	886.7	691.8	51.4	640.4
74	917.6	30.9	886.7	695.3	54.9	640.4
75	919.5	32.9	886.7	699.0	58.6	640.4
76	921.5	34.9	886.7	702.8	62.3	640.4

77	923.5	36.9	886.7	706.7	66.2	640.4
78	925.6	38.9	886.7	710.7	70.2	640.4
79	927.6	40.9	886.7	714.7	74.3	640.4
80	929.6	42.9	886.7	718.8	78.4	640.4
81	931.5	44.9	886.7	723.0	82.5	640.4
82	933.3	46.7	886.7	727.0	86.6	640.4
83	935.0	48.4	886.7	731.0	90.6	640.4
84	936.6	49.9	886.7	734.9	94.5	640.4
85	937.9	51.3	886.7	738.7	98.2	640.4
86	939.1	52.4	886.7	742.1	101.6	640.4
87	939.9	53.3	886.7	745.2	104.7	640.4
88	940.5	53.9	886.7	747.9	107.4	640.4
89	940.8	54.1	886.7	750.1	109.6	640.4
90	940.7	54.1	886.7	751.7	111.3	640.4
91	940.4	53.7	886.7	752.8	112.4	640.4
92	939.7	53.0	886.7	753.3	112.9	640.4
93	938.7	52.1	886.7	753.3	112.8	640.4
94	937.6	51.0	886.7	752.7	112.3	640.4
95	936.4	49.7	886.7	751.7	111.3	640.4
96	935.0	48.3	886.7	750.4	109.9	640.4
97	933.6	46.9	886.7	748.6	108.2	640.4
98	932.1	45.5	886.7	746.6	106.2	640.4
99	930.8	44.1	886.7	744.4	104.0	640.4
100+	929.5	42.9	886.7	742.1	101.7	640.4

IHD, ischemic heart disease

Table S7. Etiologic effects of Relative risk for IHD per 2%E from TFA.

**Age (y)**	**Relative risk (95% confidence interval)**
25-29	1.901 (1.591-2.275)
30-34	1.775 (1.514-2.085)
35-39	1.615 (1.415-1.848)
40-44	1.517 (1.352-1.707)
45-49	1.461 (1.316-1.627)
50-54	1.396 (1.274-1.535)
55-59	1.323 (1.225-1.433)
60-64	1.264 (1.186-1.352)
65-69	1.222 (1.157-1.294)
70-74	1.186 (1.132-1.246)
75-79	1.158 (1.112-1.207)
80-84	1.150 (1.107-1.197)
85-89	1.150 (1.107-1.197)
90-94	1.150 (1.107-1.197)
95+	1.150 (1.107-1.197)

IHD, ischemic heart disease; TFA, trans-fatty acids.

Table S8. Estimated policy costs of a mandatory limit of industrial trans fatty acids in the Nigerian food supply over 5 years, 10 years, and the population lifetime.

	**5 years**	**10 years**	**Population lifetime**
Total costs, millions USD	15.6 (9.8; 21.1)	17.2 (11.2; 23.0)	26.2 (19.0; 33.2)
Government costs, thousands USD	885 (795; 974)	1,960 (1,809; 2,120)	7,811 (7,540; 8,087)
National legislation	25 (16; 35)	25 (16; 35)	25 (16; 35)
NAFDAC	736 (654; 814)	1,811 (1,661; 1,963)	7,661 (7,394; 7,926)
Work force expenses	37 (31; 44)	69 (61; 78)	243 (231; 255)
Enlightenment activities	40 (24; 55)	40 (24; 55)	40 (24; 55)
Enforcement activities	309 (254; 366)	576 (500; 652)	2,024 (1,920; 2,134)
Workshops activities	122 (74; 170)	122 (74; 170)	122 (74; 170)
Monitoring	124 (104; 146)	230 (203; 259)	809 (768; 852)
Other activities e.g., research	103 (85; 121)	775 (651; 898)	4,423 (4,176; 4,650)
FMoH (legislation)	71 (44; 100)	71 (44; 100)	71 (44; 100)
SON (revising food standard regulations)	14 (9; 20)	14 (9; 20)	14 (9; 20)
CPC (enlightenment activities)	39 (24; 55)	39 (24; 55)	39 (24; 55)
Industry costs, millions USD	14.7 (9.0; 20.3)	15.3 (9.3; 21.1)	18.4 (11.2; 25.4)

CPC, Consumer Protection Commission; FMoH, Federal Ministry of Health; NAFDAC, National Agency for Food and Drug Administration and Control; SON, Standard Organisation of Nigeria.

Table S9. Estimated cost-effectiveness of implementing a mandatory limit of iTFA content (≤2% of all fats) in the Nigerian food supply, in main analysis and deterministic sensitivity analyses.

	Main analysis	Lower pre-intervention intake[2]	Higher pre-intervention intake[3]	Intake reduction: 0.05%E	Intake reduction: 0.10%E	Intake reduction: 0.15%E	Intake reduction: 0.20%E	0% discount rate[4]	6% discount rate[5]	More products reformulated[6]	No reformulation costs[7]	50% greater government costs[8]
5 years												
HALYs gained, thousands	4.52 (4.00; 5.02)	2.28 (2.03; 2.57)	6.71 (6.00; 7.45)	0.82 (0.74; 0.91)	1.64 (1.48; 1.81)	2.45 (2.23; 2.69)	3.23 (2.92; 3.54)	4.93 (4.41; 5.49)	4.14 (3.71; 4.61)	4.52 (4.00; 5.02)	4.52 (4.00; 5.02)	4.52 (4.00; 5.02)
Net costs, million USD	-18.5 (-25.7; -11.1)	-1.6 (-8.0; 4.5)	-34.9 (-43.6; -26.6)	9.31 (3.44; 15.18)	3.05 (-2.89; 8.86)	-3.15 (-9.37; 2.94)	-9.14 (-15.65; -2.66)	-20.6 (-28.2; -13.5)	-16.4 (-23.6; -9.4)	-3.7 (-15.7; 8.7)	-33.2 (-37.9; -28.7)	-17.6 (-24.9; -10.3)
ICER, USD per HALY gained	dominant (dominant; dominant)	dominant (dominant; 2,024)	dominant (dominant; dominant)	11,302 (3,991; 19,134)	1,874 (dominant; 5,516)	dominant (dominant; 1,209)	dominant (dominant; dominant)	dominant (dominant; dominant)	dominant (dominant; dominant)	dominant (dominant; 1,994)	dominant (dominant; dominant)	dominant (dominant; dominant)
Probabilities												
Cost saving	100%	69.9%	100%	0.2%	15.3%	84.9%	99.8%	100%	100%	71.6%	100%	100%
Cost-effective	100%	78.6%	100%	0.5%	20.9%	90.6%	99.9%	100%	100%	80.4%	100%	100%
10 years												
HALYs gained, thousands	24.8 (21. 9; 27.6)	12.5 (11.1; 14.1)	36.8 (32.9; 40.9)	4.52 (4.05; 4.99)	9.00 (8.12; 9.95)	13.5 (12.2; 14.8)	17.8 (16.0; 19.5)	30.1 (26. 9; 33.6)	20.5 (18.4; 22.9)	24.8 (21. 9; 27.6)	24.8 (21. 9; 27.6)	24.8 (21.9; 27.6)
Net costs, million USD	-72.4 (-85.4; -59.5)	-28.0 (-36.8; -19.9)	-115.7 (-133.6; -99.2)	0.63 (-6.13; 7.38)	-15.91 (-23.61; -8.96)	-32.31 (-40.71; -24.20)	-48.2 (-57.8; -38.7)	-84.3 (-98.8; -71.0)	-61.7 (-73.5; -50.7)	-57.0 (-74.0; -40.6)	-87.7 (-99.6; -76.0)	-70.4 (-83.5; -57.7)
ICER, USD per HALY gained	dominant (dominant; dominant)	dominant (dominant; dominant)	dominant (dominant; dominant)	127 (-dominant; 1,682)	dominant (dominant; dominant)	dominant (dominant; dominant)	dominant (dominant; dominant)	dominant (dominant; dominant)	dominant (dominant; dominant)	dominant (dominant; dominant)	dominant (dominant; dominant)	dominant (dominant; dominant)
Probabilities												
Cost saving	100%	100%	100%	42.9%	99.9%	100%	100%	100%	100%	100%	100%	100%
Cost-effective	100%	100%	100%	63.6%	100%	100%	100%	100%	100%	100%	100%	100%
Lifetime												
HALYs gained, thousands	760 (668; 851)	383 (339; 433)	1,131 (1,003; 1,263)	139 (124; 154)	276 (248; 307)	413 (373; 456)	545 (490; 599)	2,353 (2,081; 2,641)	303 (270; 341)	760 (668; 851)	760 (668; 851)	760 (668; 851)
Net costs, million USD	-492 (-561; -427)	-235 (-273; -203)	-744 (-843; -652)	-67.5 (-82.3; -54.1)	-161 (-184; -140)	-254 (-286; -223)	-345 (-388; -306)	427 (290; 568)	-311 (-353; -271)	-473 (-543; -407)	-510 (-579; -443)	-484 (-553; -419)
ICER, USD per HALY gained	dominant (dominant; dominant)	dominant (dominant; dominant)	dominant (dominant; dominant)	dominant (dominant; dominant)	dominant (dominant; dominant)	dominant (dominant; dominant)	dominant (dominant; dominant)	181 (124; 237)	dominant (dominant; dominant)	dominant (dominant; dominant)	dominant (dominant; dominant)	dominant (dominant; dominant)
Probabilities												
Cost saving	100%	100%	100%	99.2%	100%	100%	100%	0%	100%	100%	100%	100%
Cost-effective	100%	100%	100%	99.5%	100%	100%	100%	100%	100%	100%	100%	100%

HALY, health-adjusted life years; ICER, incremental cost-effectiveness ratio; iTFA, industrial trans-fatty acids; USD, United States Dollars. 1Higher post-intervention intake assumed (mean: 0.10%E, SD: 0.01%E). 2Pre-intervention intakes 50% lower compared to main analysis. 3Pre-intervention intakes 50% higher compared to main analysis. 4Assuming 0% discount rate for costs and HALYs. 5Assuming 0% discount rate for costs and HALYs. 6The number of products potentially containing iTFA assumed to be twice as many as in the main analysis. 7Reformulation was considered to be part of the natural life cycle of a product with no additional cost. No additional reformulation costs were assumed. 8Monitoring costs were assumed to be 50% greater compared to the main analysis.

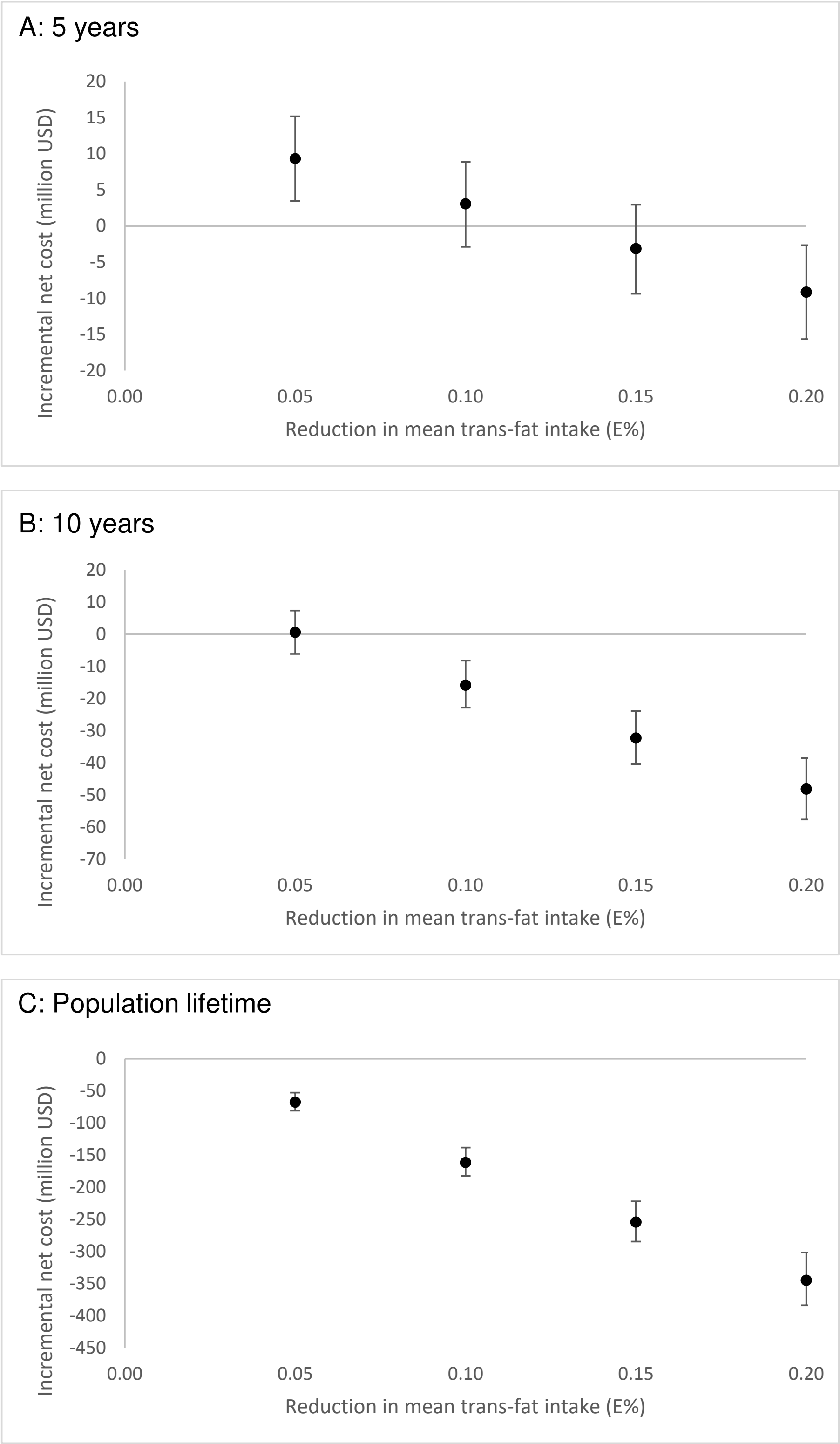

Supplementary figure S1. Net costs estimated in threshold analysis with gradually increasing intervention effects on iTFA intake (0.05-0.20%E reduction from base population intake), evaluated for diverse time horizons: 5 year (A), 10 year (B), and population lifetime (C). Dots indicate mean of n=2,000 Monte Carlo simulations, and the error bars the 95% uncertainty interval (defined as the 2.5^{th} and 97.5^{th} percentiles of model estimates).

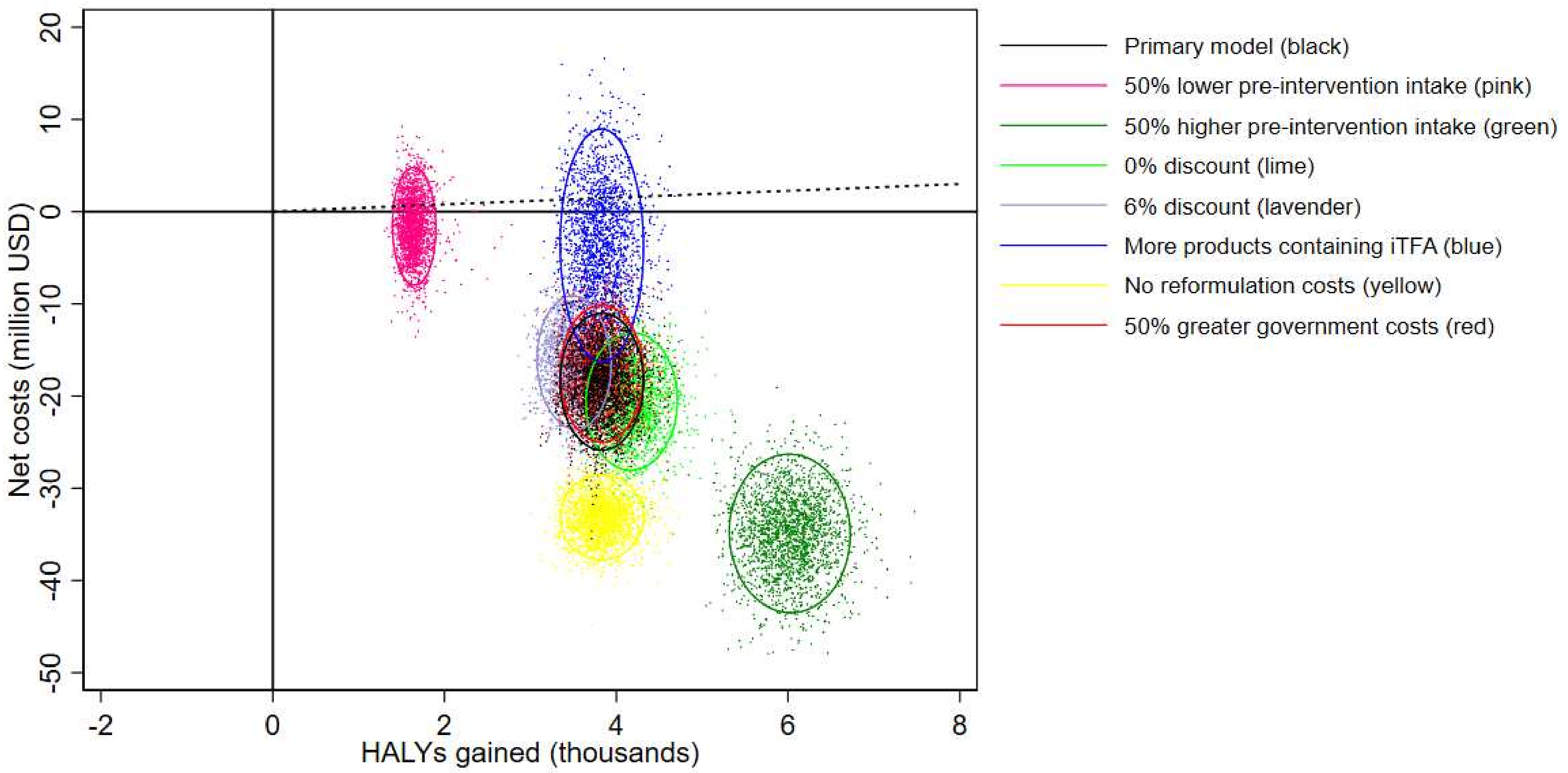

Supplementary figure S2. Net costs and health-adjusted life years (HALYs) gained during the first 5 years after policy implementation estimated in the primary model and in deterministic sensitivity analyses. Dotted and dashed black lines indicate thresholds for cost-effective and very cost-effective interventions, respectively.

References

1. Mozaffarian D, Clarke R. Quantitative effects on cardiovascular risk factors and coronary heart disease risk of replacing partially hydrogenated vegetable oils with other fats and oils. *Eur J Clin Nutr* 2009; **63 Suppl 2**: S22-33.
2. Mozaffarian D, Katan MB, Ascherio A, Stampfer MJ, Willett WC. Trans Fatty Acids and Cardiovascular Disease. 2006; **354**(15): 1601-13.
3. Wanders AJ, Zock PL, Brouwer IA. Trans Fat Intake and Its Dietary Sources in General Populations Worldwide: A Systematic Review. *Nutrients* 2017; **9**(8): 840.
4. Uauy R, Aro A, Clarke R, et al. WHO Scientific Update on trans fatty acids: summary and conclusions. *Eur J Clin Nutr* 2009; **63**: S68.
5. Downs SM, Bloem MZ, Zheng M, et al. The Impact of Policies to Reduce trans Fat Consumption: A Systematic Review of the Evidence. *Current Developments in Nutrition* 2017; **1**(12).
6. L'Abbé MR, Stender S, Skeaff CM, Ghafoorunissa, Tavella M. Approaches to removing trans fats from the food supply in industrialized and developing countries. *Eur J Clin Nutr* 2009; **63**(2): S50-S67.
7. Final Determination Regarding Partially Hydrogenated Oils. Notification; declaratory order; extension of compliance date. *Federal register* 2018; **83**(98): 23358-9.
8. Marklund M, Zheng M, Veerman JL, Wu JHY. Estimated health benefits, costs, and cost-effectiveness of eliminating industrial trans-fatty acids in Australia: A modelling study. *PLOS Medicine* 2020; **17**(11): e1003407.
9. GBD 2019 Risk Factors Collaborators. Global burden of 87 risk factors in 204 countries and territories, 1990–2019: a systematic analysis for the Global Burden of Disease Study 2019. *The Lancet* 2020; **396**(10258): 1223-49.
10. Global Burden of Disease Collaborative Network. Global Burden of Disease Study 2019 (GBD 2019) Results: Seattle, United States of America: Institute for Health Metrics and Evaluation (IHME), 2020.
11. Barendregt JJ, van Oortmarssen GJ, Vos T, Murray CJL. A generic model for the assessment of disease epidemiology: the computational basis of DisMod II. *Population Health Metrics* 2003; **1**(1): 4.
12. Salomon JA, Haagsma JA, Davis A, et al. Disability weights for the Global Burden of Disease 2013 study. *The Lancet Global Health* 2015; **3**(11): e712-e23.
13. Rosendaal NTA, Hendriks ME, Verhagen MD, et al. Costs and Cost-Effectiveness of Hypertension Screening and Treatment in Adults with Hypertension in Rural Nigeria in the Context of a Health Insurance Program. *PLOS ONE* 2016; **11**(6): e0157925.
14. Hendriks ME, Bolarinwa OA, Nelissen HE, et al. Costs of cardiovascular disease prevention care and scenarios for cost saving: a micro-costing study from rural Nigeria. *J Hypertens* 2015; **33**(2): 376-684.
15. The World Bank Group. Inflation, Consumer prices (annual %)- Nigeria. 2022. https://data.worldbank.org/indicator/FP.CPI.TOTL.ZG?locations=NG.
16. Federal Ministry of Health. National Health Accounts 2017. 2019.
17. Ministry of Health. 2013 Kenya Household Health Expenditure and Utilisation Survey. Nairobi, Kenya: Government of Kenya; 2014.
18. Huang L, Ojo AE, Kimiywe J, et al. Presence of trans-Fatty Acids Containing Ingredients in Pre-Packaged Foods and the Availability of Reported trans-Fat Levels in Kenya and Nigeria. *Nutrients* 2023; **15**(3): 761.
19. Allen K, Pearson-Stuttard J, Hooton W, Diggle P, Capewell S, O'Flaherty M. Potential of trans fats policies to reduce socioeconomic inequalities in mortality from coronary heart disease in England: cost effectiveness modelling study. *BMJ* 2015; **351**: h4583.
20. Pearson-Stuttard J, Hooton W, Critchley J, et al. Cost-effectiveness analysis of eliminating industrial and all trans fats in England and Wales: modelling study. *Journal of public health (Oxford, England)* 2017; **39**(3): 574-82.
21. Barendregt JJ, Veerman JL. Categorical versus continuous risk factors and the calculation of potential impact fractions. *Journal of epidemiology and community health* 2010; **64**(3): 209-12.
22. Afshin A, Sur PJ, Fay KA, et al. Health effects of dietary risks in 195 countries, 1990-2017: a systematic analysis for the Global Burden of Disease Study 2017. *The Lancet* 2019.
